# The Sill beneath the Door

**Published:** 1863-10

**Authors:** 


### THE SILL BENEATH THE DOOR.

BY HARBAUGH.

THERE is a strange, a mystic spell
Of memory and love,
That chains my heart to early days,
Where'er I rest or rove.
I see again the old home house,
I walk across each floor;
I go the passage through, and stand
With farewell words and staff in hand,
Upon the sill
That lies beneath the door.

Each spot around that dear old home,
Its well-kept treasure gives:
In every tree, and wall, and chair,
Some cherished memory lives;
But no where beats my heart so high,
And no where feel I more
Than here, when musingly I stand,
With farewell words and staff in hand,
Upon the sill
That lies beneath the door.

What silent years have fled since I
Looked out from dear old home,
With hopeful heart, through moist'ning eye,
For better days to come!
'Twas here I turned to those I left,
With longing heart once more—
Here lingered still, where now I stand
With farewell words and staff in hand,
Upon the sill
That lies beneath the door.

I've passed o'er other thresholds since,
To grander halls—but still
I never entered home like this,
Across another sill.
Parents and home we have but once,
When gone they come no more!
Oh! what a moment when we stand,
With farewell words and staff in hand,
Upon the sill
That lies beneath the door.

